# Effect on osseointegration of dental implants treated with carboxyethylphosphonic acid and functionalized with BMP-2: preliminary study on a minipig model

**DOI:** 10.3389/fbioe.2023.1244667

**Published:** 2023-07-27

**Authors:** Nansi López-Valverde, Javier Aragoneses, Cinthia Rodríguez, Juan Manuel Aragoneses

**Affiliations:** ^1^ Department of Surgery, Faculty of Medicine, Instituto de Investigación Biomédica de Salamanca (IBSAL), Universidad de Salamanca, Salamanca, Spain; ^2^ Department of Medicine and Medical Specialties, Faculty of Health Sciences, Universidad Alcalá de Henares, Madrid, Spain; ^3^ Department of Dentistry, Universidad Federico Henríquez y Carvajal, Santo Domingo, Dominican Republic; ^4^ Faculty of Dentistry, Universidad Alfonso X El Sabio, Madrid, Spain

**Keywords:** dental implants, carboxyethylphosphonic acid, BMP-2, osseointegration, minipig model

## Abstract

**Introduction:** Rough titanium surfaces biofunctionalised by osteogenic proteins, such as BMP-2, have been shown to accelerate the osseointegration process and reduce waiting times for prosthetic loading. The preclinical study presented here compared the bone in contact with the implant and bone neoformation and density between titanium (Ti) implants with a conventional etched surface (SLA type) and others treated with carboxyethylphosphonic acid (CEPA) and bone morphogenetic protein 2 (BMP-2), after 4 weeks of implantation in the tibia of a minipig model.

**Methods:** Sixteen implants (eight experimental and eight control) of Ti-Al16-V4 with a tapered screw design and internal hexagonal connection were randomly inserted into the tibiae of four minipigs, four in each tibia. The experimental implants were treated with CEPA and BMP-2 and sterilised with gamma radiation (25 KG). The insertion torque was 40 N and primary stability was measured with the Osstell^®^ device (ISQ 64 ± 2.6). Five bone parameters were evaluated: bone in contact with the implant (BIC), bone in contact with the corrected implant (BICc), new bone formation (BV/TV), bone density between threads (BAI/TA) and peri-implant bone density (BAP/TA). A histomorphometric study was performed and the samples were digitised with Adobe Photoshop Cs6. Statistical analysis of the variables was performed using SAS 9.4.

**Results:** After a period of 4 weeks, no significant clinical signs were observed and all implants were integrated. Light microscopy of the experimental group revealed an ICB with no signs of fiber tissue, but with areas of ectopic new bone in the medullary space. Statistical analysis showed significant results for BIC and BICc (*p* = 0.0001 and *p* = 0.001, respectively). No statistical signification was found for the other parameters evaluated.

**Conclusion:** Despite the limitations of this study, our results demonstrated that dental implant surfaces treated with CEPA and BMP-2 improve their biological response to osseointegration.

## 1 Introduction

Since Per-Ingvar Brånemark in 1969 demonstrated the long-term success of Ti dental implants and André Schroeder in 1976 introduced the rough surface, both macro- and micro design have undergone profound transformations (Brånemark et al., 1969; Schroeder et al., 1976).

In 1990 (Wilke et al., 1990) published the first *in vivo* study on acid-etched sandblasted implants (SLA). Subsequently, multiple investigations have studied its biological properties (Cochran et al., 1998; Buser et al., 1999; Cochran et al., 2002; Li et al., 2002).

Ti and its alloys, despite their good biocompatibility, require long periods of time to ensure their osseointegration. To accelerate and ensure their osseointegration, different surface coating methods have been used, such as hydroxyapatite (HA) or calcium phosphate (Ca/P), although these types of coatings have certain limitations and cannot be applied to implants with porous surfaces, nor to those in which it is desired to incorporate biological molecules into the coating (de Groot et al., 2011). In addition, dental implants require biocompatibility with soft tissues, to achieve adhesion of the gingival epithelium and certain antibacterial properties that prevent the formation of biofilms (Hanawa, 2010).

The acid etching process, besides increasing roughness and cleaning the surface, it has been demonstrated that this type of etching increases cell adhesion and bone neoformation, improving osseointegration (Cho and Park, 2003; Stepanovska et al., 2020). Some studies have shown that dual acid treatments will improve osseointegration, since the surface achieved will largely mimic the bone structure (Jang et al., 2017; Giner et al., 2018).

Phosphonic acids are organophosphorus compounds, poorly soluble in water, which have been used, due to their bioactive properties, for surface functionalization, in particular to immobilize organic molecules on the surface of metal oxides such as Ti oxide (TiO_2_) (Zeininger et al., 2016; Sevrai et al., 2017). Some investigations have used phosphonic acid molecules for the formation of self-assembled monolayers (SAM) on the Ti surface (Gawalt et al., 2001; Adden et al., 2006) that would facilitate the immobilization of bioactive molecules, such as BMP-2, with the capacity to differentiate a good number of mesenchymal cells into osteoblasts and, therefore, generate increased bone growth at the local level (Hoffmann et al., 2001). BMP-2 is considered a potent osteogenic growth factor, capable of promoting cell differentiation, inducing bone healing by recruiting bone-forming cells and providing biomaterials with enhanced osteoinductive capacity (Chen et al., 2004). Currently, it has two FDA-approved indications: treatment of unconsolidated tibial fractures and treatment of open tibial fractures treated with intramedullary fixations (Ronga et al., 2013). Some studies have evaluated its osteoinductive properties in critical defects in different experimental models, reporting high osteoblastic activity and physiological bone regeneration at the experimental sites (von Walter et al., 2008; Kolk et al., 2019). In addition, it has been described that it would act as an accelerator of osteoblastic differentiation, giving rise to a new bone matrix, which would include mineral precursors and collagen (de Queiroz Fernandes et al., 2018). All this demonstrates that BMP-2 can potentially be applied in peri-implant bone regeneration and that it would have the capacity to reduce waiting times, while guaranteeing osseointegration.

The objective of our research was to analyze and compare the effect of carboxyethylphosphonic acid (HO_2_C-CR_1_H-CR_2_H-PO_3_H_2_) (CEPA) and bone morphogenetic protein (BMP-2) on the osseointegration of dental implants placed in an experimental animal model, based on the null hypothesis (H_0_) that there are no differences in the histomorphometric parameters related to osseointegration between dental implants with surfaces treated with CEPA and BMP-2 and implants with SLA surfaces.

## 2 Material and methods

### 2.1 Study design; implants design; study animals

**FIGURE 1:**
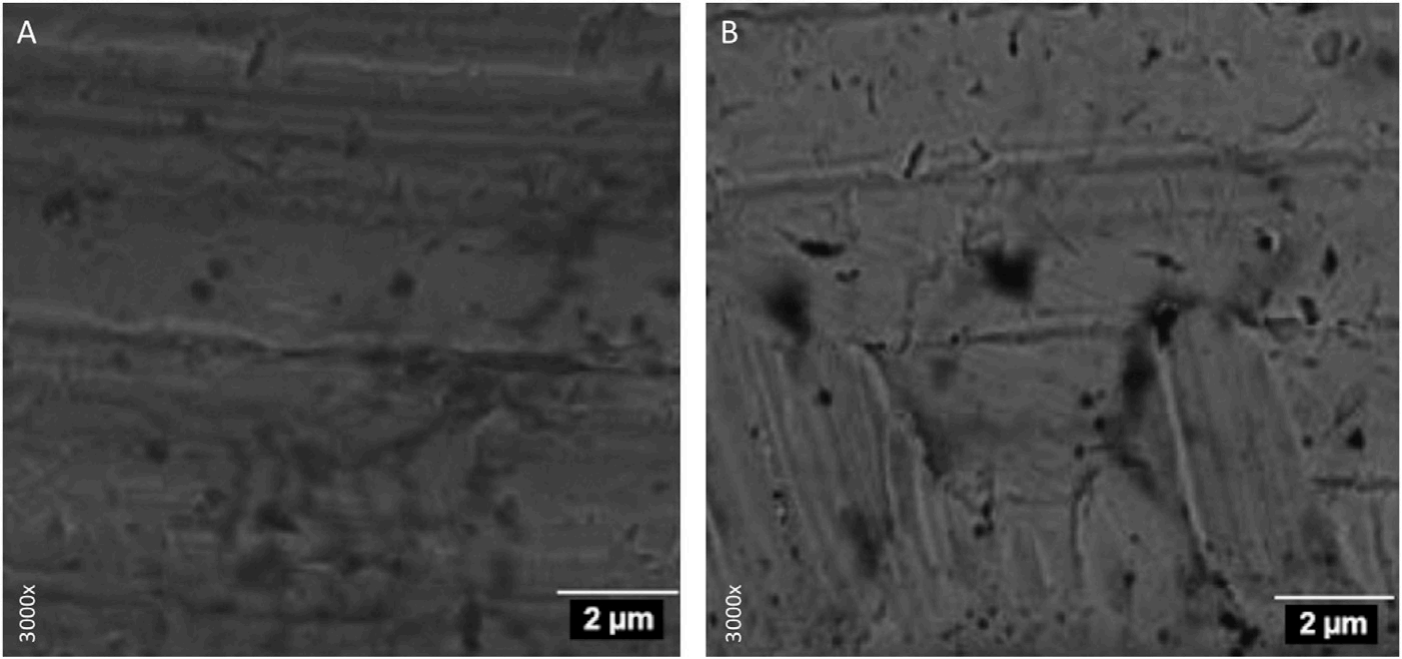
Electron microscopy. Implant platform surface: **(A)** belonging to the control group and **(B)** treated with carboxylphosphonic acid.

The study protocol consisted in randomly inserting 16 conical implants (four in each tibia of each animal), manufactured with grade IV Ti (90wt% Ti, 6wt% Al and 4wt% V) of 4 mm Ø × 10 mm length, with internal connection hexagon and conventional treatment of the implant surface, by coarse-grained sandblasting and acid etching (SLA), in the tibiae of 4 minipigs. Two study groups were established, with 8 implants in each group: the experimental group with CEPA-treated implants functionalized with BMP-2 and the control group with SLA-surfaced implants. The implants of the experimental group received surface treatment by CEPA (Figure 1.) and BMP-2, according to the method proposed by Aresti et al. (2021). For this purpose, they were subjected to a dilution of 50 mL of tetrahydrofuran (THF) (Uvasol^®^, Madrid, Spain) and 55 mg of CEPA for 1 day at 76°C. Subsequently, CEPA was activated with a solution of 5 mL of distilled H_2_O, 175 mL of ethyl-3-(3-dimethylaminopropyl) carboxyamide (EDC) and 54 mg of N-hydroxysulfamide (NHS) for 15 min at room temperature. The pH stability (pH 7) was checked using a pH-meter (MP230, Mettler Toledo^®^, Barcelona, Spain) during the whole process. EDC activates the carboxyl and amine groups, reacting with the carboxyl group to form an O-acylisourea intermediate; however, if it does not react with the amine, it hydrolyzes and regenerates the carboxyl group, thus incorporating NHS. In the presence of NHS, EDC can be used to convert the carboxyl groups to amine-reactive NHS esters by activating CEPA with EDC and NHS to react with the amino groups of BMP-2. Once the carboxyl groups were activated, 20 mg of BMP-2 was incubated for 1 h at 37°C. To remove impurities, the implants were exposed to ultrasonic waves and packed in laminar flow cabinets in sterile atmosphere; they were sterilized by 25 KGy gamma radiation. The implants were concealed from the operator by means of a depistage wrap.

Four Landrace Large White minipigs between 18 and 20 weeks of age were selected for the study in accordance with the European Committee for Standardization guidelines for bone tissue studies. The randomized experimental investigation was approved on 31 January 2013 by the Animal Experimentation Ethics Committee (CEEA) of the Hospital Universitario Puerta de Hierro Majadahonda (Madrid, Spain) and the Instituto de Investigación Puerta de Hierro Majadahonda, with CEEA Code: 017/2013. The study was conducted in accordance with the ethical principles of the ARRIVE guidelines on animal experimentation.

### 2.2 Surgeries; euthanasia procedure

General anesthesia, performed by a specialized veterinarian, was induced with 0.2–0.4 mg/kg propofol intravenously (Diprivan^®^, AstraZeneca, Cambridge, United Kingdom), using a 20 G needle and epidural anesthesia with bupivacaine (Bupinex^®^, Richmond Vet Pharma, Buenos Aires, Argentina) and fentanyl (Fentanyl^®^, Kilab, Buenos Aires, Argentina). In addition, local anesthesia was infiltrated in the surgical area with 4% articaine and epinephrine 1:200,000 (Ultracain^®^, Normon, Madrid, Spain). A N°7 endotracheal tube with a balloon cuff was placed and connected to a circular anesthesia circuit (Leon Plus, Heinen&Löwenstein, Bad Ems, Germany). Multimodal analgesia was used during the study, using medetomidine 0.01 mg/kg (Medetor^®^, Virbac, Carros, France), ketamine 5.0 mg/kg (Ketonal 50^®^, Richmond Vet Pharma, Buenos Aires, Argentina), midazolam 0.2 mg/kg (Dormicum^®^, Roche S.A., Basel, Switzerland) and atropine 0.02 mg/kg (Atropina^®^, Pharmavet, Bogotá, Colombia).

**FIGURE 2:**
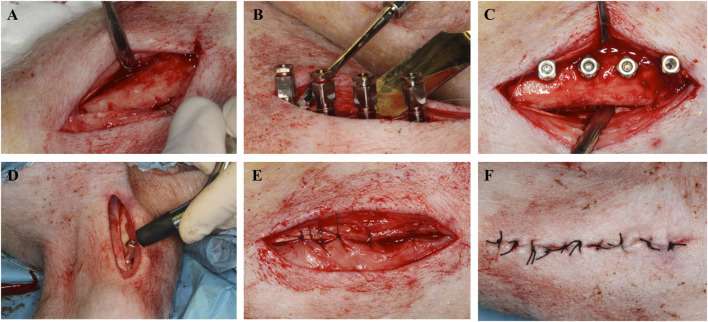
**(A–F)** Surgical images.

Next, after making an incision in the anterior aspect of the tibia (Figure 2A), a drilling sequence was performed, according to the implant manufacturer’s recommendations, using a micromotor (AM-25 E RM, W&H, Bürmoos, Austria) and a 20:1 reduction contra-angle handpiece (WS-75 LG, W&H, Bürmoos, Austria) with profusion of saline (Vitulia^®^ 0.9%, Barcelona, Spain). Subsequently, four implants, with their corresponding locking screws, were placed in the tibial crest of each animal (Figures 2B, C), using the randomization program Surgimplant IPX, (Galimplant, Sarria, Lugo, Spain). The placement torque was 40N with a primary stability ISQ 64 ± 2.6 (Osstell^®^ device, Ostell/Integration Diagnostics, Gothenburg, Sweden) (Figure 2D).

The incisions were closed by simple stitches in two planes: in the deep plane a polyglactin rapid resorbable suture (5/0) was used and in the superficial plane a non-resorbable braided silk (3/0) was used (Figures 2E, F).

The animals were euthanized randomly, 4 weeks after surgery, by overdose of sodium pentobarbital, under premedication administered intramuscularly, with Zolacepam-Tiletamine-Medetomidine (Zoletil 5 mg/kg, Medetonin 0.1 mg/kg).

### 2.3 Histomorphometric analysis

**FIGURE 3:**
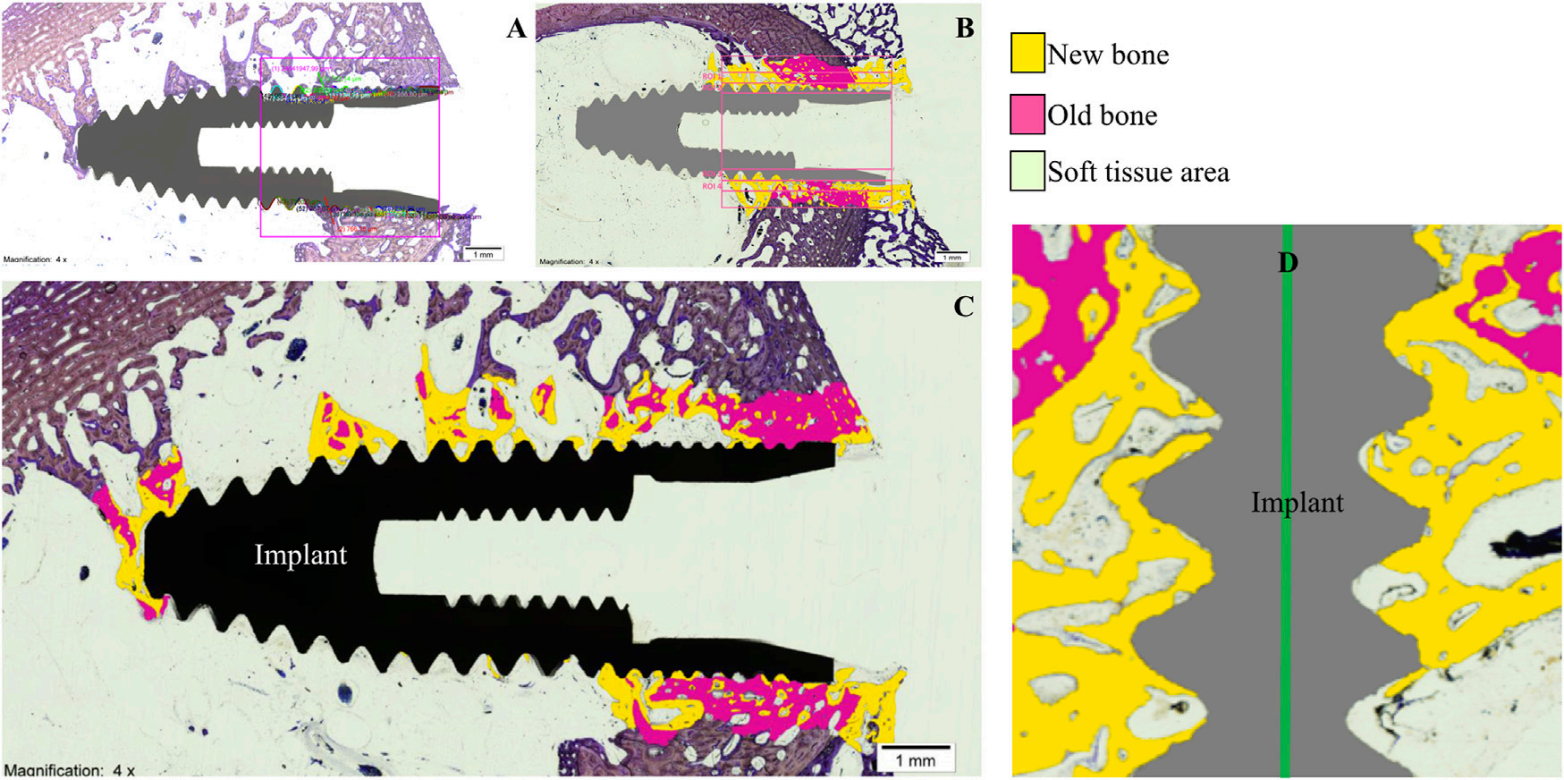
**(A)**, area of interest; **(B)**, regions of interest; **(C)**, digitized image; **(D)**, new bone in contact with the implant surface.

Implant-bearing tibiae were dissected and extracted and fixed in 10% buffered formalin solution for 2 weeks to allow histomorphometric processing. Subsequently, bone-implant fragments were extracted with an oscillating saw in 16 mm thick serial sagittal sections and dehydrated in increasing alcohol solutions (80, 96%, and 100%) for 3 days. The samples were embedded in glycolmethacrylate (GMA; 2-hydroxyethyl methacrylate, HEMA, JB-4; JB-4 Plus) (Technovit 7200 VLC, Heraeus Kulzer, Wehrheim, Germany). Finally, they were sectioned into 50 µm thick slices, stained with the Levai Laczko staining technique (Kuchler et al., 2013) and examined with light optical microscopy (BX51, Olympus, Tokyo, Japan) by an experienced pathologist blinded to the randomization of the study groups. In addition, histological images were processed, digitized and loaded into a computer program (Adobe Photoshop Cs6, San Jose, CA, USA; Cell Sens Dimensions, Olympus, Tokyo, Japan) to evaluate, on a percentage basis, 5 parameters, within an area of interest (AOI) of 5 mm × 5 mm. For the calculation of densities, regions of interest (ROI) were established with a thickness of 300 microns, drawn parallel to the peak of the implant threads (Figures 3A, B). The Adobe Photoshop Cs6 program encoded in a digitizer tablet, the bone surrounding the implants, in yellow color the new bone and in pink color the old bone (Figure 3C). The parameters evaluated were:

- Bone-to-implant contact (BIC): percentage of the dental implant surface in contact with the surrounding bone.
- Corrected bone-to-implant contact (BICc): length of bone in contact with the surface of the dental implant, excluding regions not covered by bone.
- New bone formation (BV/TV): area of new bone formed after dental implant placement.
- Bone density between threads (BAI/TA): area of threads covered by surrounding bone.
- Peri-implant bone density (BAP/TA): area of bone growing along the implant.

### 2.4 Statistical test

Statistical analysis of all variables was performed with SAS 9.4 (SAS Institute Inc., Cary, NC, United States). Descriptive statistics were expressed as means, medians and standard deviations (SD) for quantitative variables. Comparative analysis was performed by comparing the histomorphometric parameters BIC (%), BICc (%), BV/TV (%), BAI/TA (%) and BAP/TA (%) between the SLA and BMP-2 study groups, using Student’s t-test and the nonparametric Mann-Whitney test. Shapiro-Wilk test was used to contrast the normality of data. Statistical significance was set at *p* < 0.05.

## 3 Results

### 3.1 General clinical observations

Sixteen 4 mm Ø x 10 mm long implants were placed in the tibial crest of 4 minipigs. All animals recovered well and after 4 weeks, at the end of the study, wound healing was uneventful, with no signs of infection, inflammation or significant subcutaneous seroma; keloid scarring was only observed in 2 of the animals used. All implants were integrated, so a total of 16 implants were available for evaluation.

### 3.2 Histomorphometry

**FIGURE 4:**
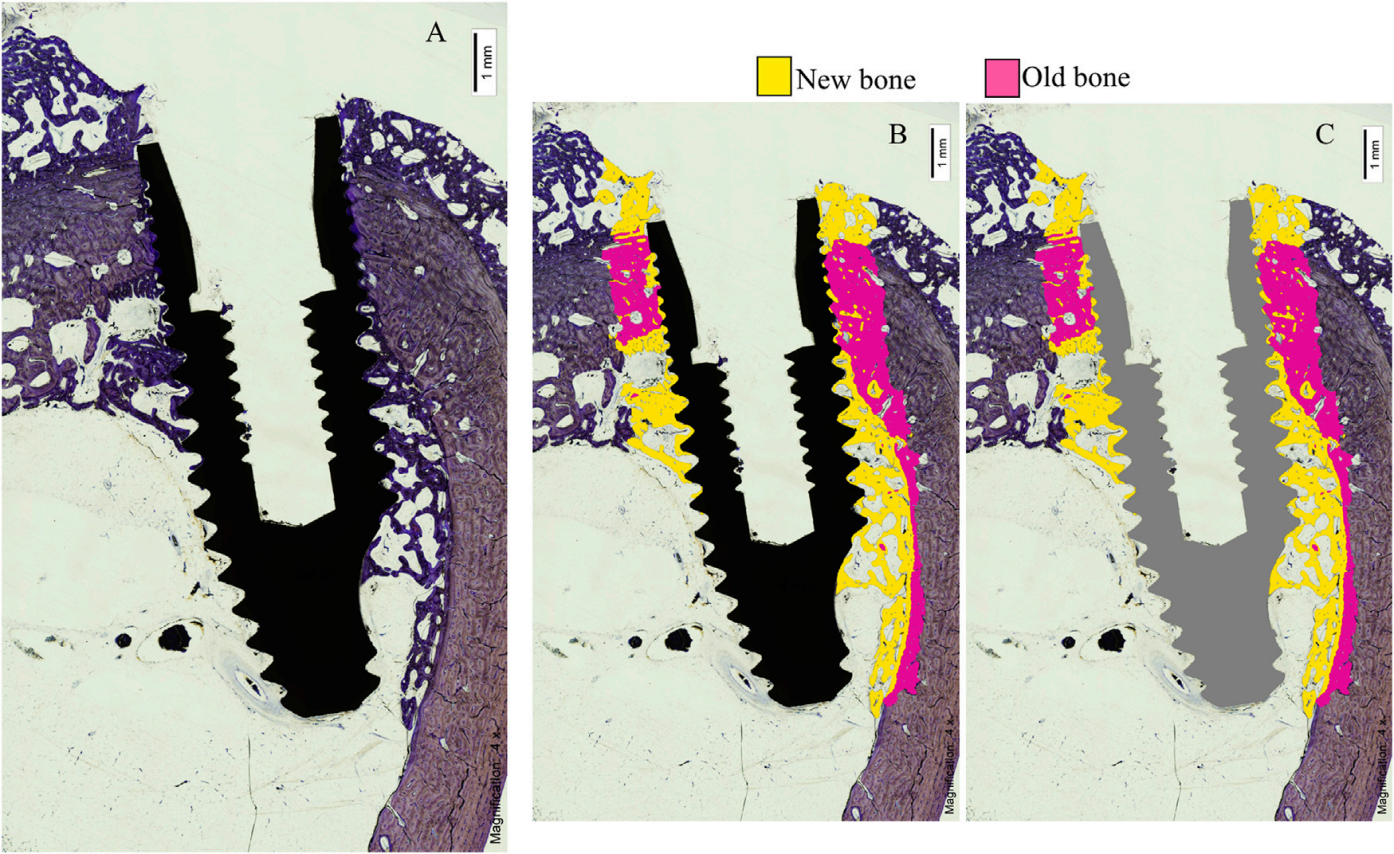
**(A)**, Histologic section with Levai Laczko stain; **(B,C)**, digitized images.

Histological analysis by light microscopy of the longitudinal sections of the samples from the experimental group revealed a BIC with interrupted medullary spaces at the bone-implant interface, but no signs of fibrous tissue (Figure 3D). The images with the implant in black show the longitudinal section of the specimen before processing by the software. The frames with the implant in gray distinguish, using the software, the areas of old bone (in pink), the areas of new bone (in yellow) and the areas of soft tissue (colorless) (Figure 3C). The experimental implants, unlike the control group, presented more areas of ectopic new bone in the medullary space (Figure 4).

### 3.3 Statistical results

**FIGURE 5:**
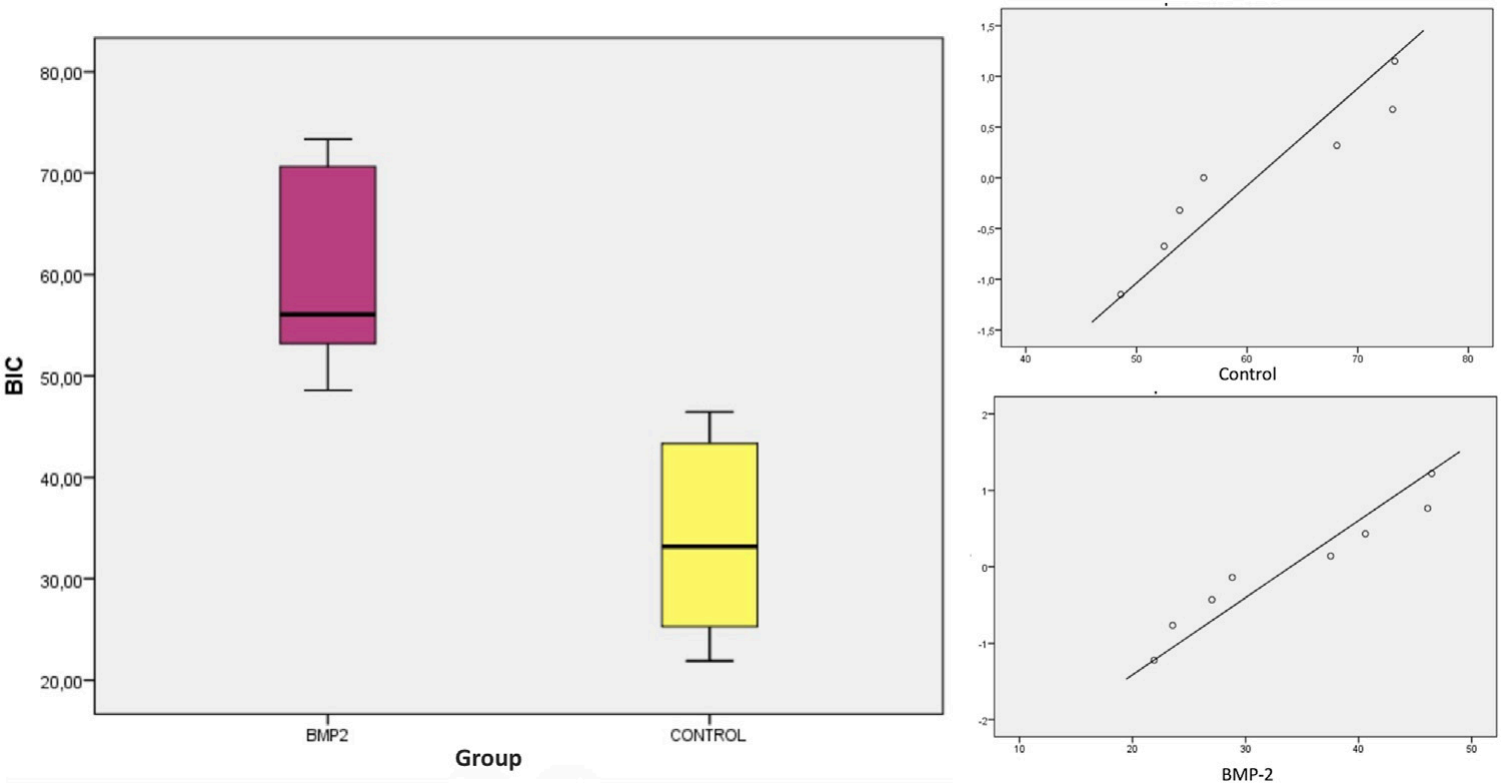
Boxplot and distribution graph of BIC values.

**FIGURE 6:**
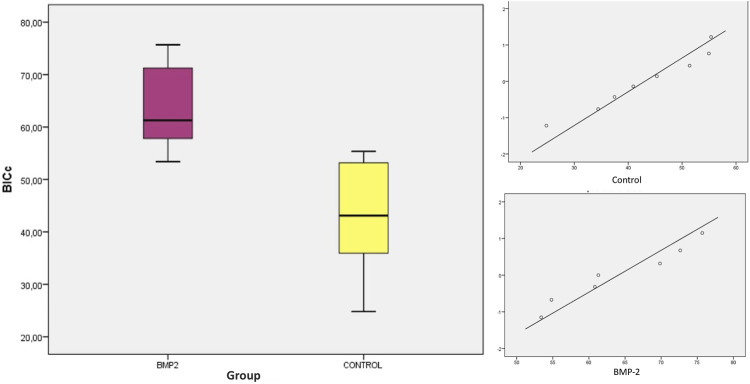
Boxplot and distribution graph of BICc values.

Using a 95% confidence interval, with a significance level <0.05, the means, medians and SD were evaluated. Student’s t-test and the nonparametric Mann-Whitney test showed statistically significant differences between groups for the variables BIC (SLA 34.001% ± 9.921% and BMP-2 60.807% ± 10.419%; *p* = 0.0001) and BICc (SLA 43.085% ± 10.766% and BMP-2 64.070% ± 8.754%; *p* = 0.001). Both variables presented a normal distribution of data (Tables 1, 2; Figures 5, 6). The variables BV/TV, BAI/TA and BAP/TA did not show statistical significance between the study groups (*p* = 0.420, *p* = 0.644 and *p* = 0.286, respectively) (Table 3).

**TABLE 1:** Descriptive statistics of the BIC (%) histomorphometric parameter.

BIC	N	Mean	Median	SD	Minimum	Maximum	*p*-value
SLA	8	34.001^a^	33.175	9.921	21.91	46.47	0.0001
BMP-2	8	60.807^b^	56.070	10.419	48.58	73.34	

Superscripts ^a^ and ^b^ refers to statistically significant differences between groups (*p* < 0.05) for Student’s *t*-test and Mann-Whitney test.

**TABLE 2:** Descriptive statistics of the BICc (%) histomorphometric parameter.

BICc	N	Mean	Median	SD	Minimum	Maximum	*p*-value
SLA	8	43.085^a^	43.130	10.766	24.81	55.37	0.001
BMP-2	8	64.070^b^	61.300	8.754	53.39	75.69	

Superscripts ^a^ and ^b^ refers to statistically significant differences between groups (*p* < 0.05) for Student’s *t*-test and Mann-Whitney test.

**TABLE 3:** Descriptive statistics of BV/TV (%), BAI/TA (%) and BAP/TA (%) histomorphometric parameters.

BV/TV (%)	N	Mean	Median	SD	Minimum	Maximum	*p*-value
SLA	8	29.767^a^	29.740	9.432	16.38	45.38	0.420
BMP-2	8	26.875^a^	31.560	7.670	15.72	34.10	
BAI/TA (%)	N	Mean	Median	SD	Minimum	Maximum	*p*-value
SLA	8	32.913^a^	34.140	7.763	22.52	44.28	0.644
BMP-2	8	30.630^a^	29.190	10.884	17.47	47.62	
BAP/TA (%)	N	Mean	Median	SD	Minimum	Maximum	*p*-value
SLA	8	44.796^a^	45.670	8.657	29.36	57.30	0.286
BMP-2	8	49.714^a^	47.700	8.387	38.00	60.86	

^a^ superscript refers to non-statistically significant differences between groups (*p* > 0.05).

## 4 Discussion

This study compared the effect on osseointegration between implants treated with carboxyethylphosphonic acid and BMP-2 and implants with conventional SLA surface in an extraoral minipig tibia model. The results showed that the experimental implants had significantly higher osseointegration parameters (BIC and BICc) than the implants with SLA surface, although the BV/TV, BAI/TA and BAP/TA values did not differ from the control group.

The ability of phosphonic acids to immobilize bioactive organic molecules on the surface of metal oxides, such as TiO_2_, confers them specific qualities that make them suitable for the fixation of osteogenic proteins such as BMP-2; moreover, nanotechnology makes possible the self-assembly of monolayers, by means of affordable processes and without the need to resort to multiple and complex techniques (Kumar et al., 2020). A study by Lan et al. (2020) observed that TiO_2_ surfaces modified with phosphonic acids had an improved hydrophilic surface that would favor biocompatibility, bone healing and osseointegration. In addition, it has been shown that surface treatment with CEPA increases surface roughness (24) and that the surface texture of an implant strongly influences the biomechanical attachment of Ti to bone (Rabel et al., 2020; Rabel et al., 2021).

The application of BMP-2 on Ti surfaces to stimulate local bone formation has been investigated in ectopic and orthotopic rodent and canine models (Liu et al., 2005; Wikesjö et al., 2008a; Geuze et al., 2012). Hall et al. (2007) observed significant bone formation around porous TiO_2_ discs coated with BMP-2 at 2 weeks after implantation, proportional to surface porosity, due to increased retention of the protein on the implant surface. Similarly, Liu et al. (2007a) observed that the osteoconductivity of the implant was severely compromised when the protein was directly adsorbed by the untreated surfaces and it has been shown that osteogenic molecules are better adsorbed by the treated surfaces, due to the slow release as the inorganic layer undergoes a degradation process (Liu et al., 2005). Coinciding with these results, our investigation found a significant difference in the BIC in experimental implants treated with BMP-2, compared to the implants of the control group with SLA surface. Liu et al. (2007b) based the osteoinductive efficacy of BMP-2 coatings on the different modes of protein release, highlighting the suitability of surfaces that mimic the mineralized bone matrix and demonstrating that the efficacy of BMP-2 would be directly related to its different initial release, depending on surface roughness. On the other hand, our study found no differences in peri-implant bone density in the experimental implants with biofunctionalized surfaces with respect to the control group, and in this aspect, (Decker et al., 2010) observed that high concentrations of BMP-2 on porous TiO_2_ implants, after a healing period of 8 weeks, gave rise to immature bone formations and decreased bone density, while low concentrations generated better bone maturation. However, there are discrepancies among researchers; studies in non-human primates, on the contrary, observed enlarged peri-implant bone remodeling zones with increasing concentrations of BMP-2 (Wikesjö et al., 2008b); moreover, we have not found unanimity in this regard on the ideal amounts of BMP-2 for implant biofunctionalization.

Nevertheless, we believe that the differences in bone density and new bone formation, could be attributed to the release kinetics of the implant surfaces and other unknown reasons.

In our research we observed that a high percentage of the samples analyzed (78%) showed ectopic bone formations in the medullary space, and in this regard, (Liu et al., 2007a) highlighted that BMP-2 has a higher osteogenic efficacy when incorporated into a carrier that mimics the mineralized bone matrix, such as the dual-etch surfaces in our study, and that it would decrease its osteogenic efficacy when incorporated on polished Ti surfaces.

The results of our study, although partially, rejected the H_0_, which assumed that there were no differences in histomorphometric parameters related to osseointegration between dental implants with conventional surfaces and those treated with CEPA and BMP-2, however, we found a series of limitations that we wish to highlight: on the one hand, the small sample size; on the other hand, our experimental model, although considered a predictive model and validated by numerous studies for the investigation of bone regeneration, has the disadvantage of being an orthotopic model with the disadvantages that this entails in terms of bone remodeling. Another question we wish to raise is the quantity and stability of the protein on the textured surface of the Ti and the time required for its effectiveness.

Therefore, we consider that more preclinical studies, appropriately designed and coincident in the methodology, investigating the osteoinductive properties of this protein, in relation to implant osseointegration would be justified and necessary.

## 5 Conclusion

Within the limitations of this study, our results demonstrated that porous surfaces of dental implants treated with CEPA and BMP-2 enhance the biological response. BMP-2 concentrations could influence bone density and bone formation.

## Data Availability

The original contributions presented in the study are included in the article/Supplementary Material, further inquiries can be directed to the corresponding author.
